# Supplementary material for: Learning curve and surgical outcome of robotic assisted colorectal surgery with ERAS program
Source: Sci Rep. 2022 Nov 29;12:20566. doi: 10.1038/s41598-022-24665-w (PMC9709162; doi:10.1038/s41598-022-24665-w)
Supplement: Supplementary file 2 — Supplementary Information 2. [file 41598_2022_24665_MOESM2_ESM.zip › RAL Raw data-Ñ[▒K/060/OP_ñw╝╨░O▒Kñσ.pdf]

正式摘要(電子簽章：000000209740952)

請選擇...reporter

臺中榮民總醫院

# Operation Note

[redacted]

- 手術日期：2021/02/05
- 手術醫師：蔣鋒帆，CR 楊曙榮，R 林志安，R 蘇少綱
- Anesthesia : GE
- Position : Lithotomy
- Aseptic agents : Soap , Betadine Alcohol , Betadine Aqua

## Diagnosis

- Pre-operative diagnosis : Malignant tumor of rectum,L/3
- Post-operative diagnosis : Ditto.

## Surgical Indication

- TUMOR RESECTION

## Operation

- Robot Assisted Low Anterior Resection + loop ileostomy

## Finding

- 1. ulcerative tumor, around 2x3 cm in size about 6 cm FAV.
  2. Total meso excision was complet
  3. IMA high ligation+
  4. distal margin 1 cm, Surefrom 45 *2 for rectum
  5. air tie test neg. donut intact.
  6. loop ileostomy in RLQ

## Procedure

- 1. under GE, the pt was in lithotomy position
  2. the trocar hold was made in periumbilicus open method. the other 5 hole made by punctiure
  3. identify holly plain and dissct. isolated IMA root. ligated with clips, so is the IMV
  4. dissect between Gerota fascia and meso rectum. lysis Lt white line
  5. dissect into pelvis area. dissect posterior side of rectum down to pelvis follor levetar ani. anterior side of Denovillor fascia. Total meso excision was complet
  6. resected rectum stump with Sure Form 45 2 fires.
  7. pull the specimen outside abdomen from periumbilical wound
  8. resect Rectum
  9. embeded envril 29 mm
  10.reset laparoscopy and complete anastomsis. air tie test neg. donut intact.
  11. loop ileostomy in RLQ
  12.she tolerate whole procedure and extubated.
- Estimated blood loss: minimum
- 器械數量: 正確
- 紗布數量: 正確

## Image

- PAINT
  - abdomen

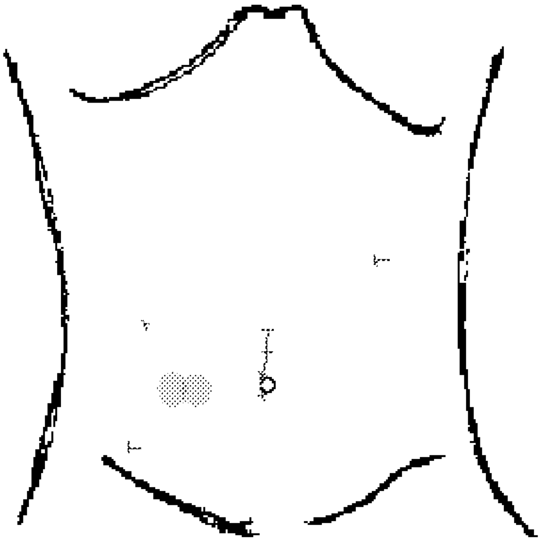

- colon & rectum

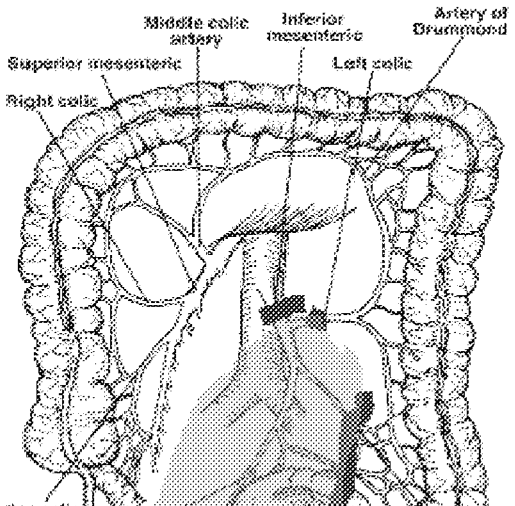


主任 2062F蔣鋒帆 / 醫師 2292G楊曙榮

正式摘要**(電子簽章：000000209740952)**

請選擇...reporter

doctorRank:B
